# “Spider Web”-like zonular deposits in pseudoexfoliation syndrome: endoscopic insights—a case report

**DOI:** 10.3389/fmed.2025.1589973

**Published:** 2025-10-01

**Authors:** Bin Lin, Ling Zhang, Dong-kan Li

**Affiliations:** ^1^School of Medicine, Xiamen Eye Center and Eye Institute of Xiamen University, Xiamen, China; ^2^Department of Ophthalmology, The Second Affiliated Hospital of Fujian Medical University, Quanzhou, Fujian, China; ^3^Xiamen Clinical Research Center for Eye Diseases, Xiamen, Fujian, China; ^4^Xiamen Key Laboratory of Ophthalmology, Xiamen, Fujian, China; ^5^Fujian Key Laboratory of Corneal & Ocular Surface Diseases, Xiamen, Fujian, China; ^6^Xiamen Key Laboratory of Corneal & Ocular Surface Diseases, Xiamen, Fujian, China; ^7^Translational Medicine Institute of Xiamen Eye Center of Xiamen University, Xiamen, Fujian, China

**Keywords:** pseudoexfoliation syndrome, endoscopic examination, zonular, pathogenesis, glaucoma

## Abstract

Pseudoexfoliation syndrome (PEX) is a systemic fibroproteinopathy with limited global epidemiological data and unclear pathogenesis. This case report describes a 70-year-old Asian woman presenting with left eye soreness and blurred vision. She was diagnosed with PEX based on typical ocular characteristics, including grayish-white debris on the pupillary margin, pigment residues in the anterior chamber angle, and annular opacity around the anterior lens capsule after pupil dilation. During surgery, endoscopic observation revealed extensive grayish-white flocculent deposits covering the entire circumference of the zonules, resembling a spider web—findings consistent with the “fibrin-like deposits” described in autopsy studies. Such exfoliated material may damage the intraocular environment through mechanical damage and chronic inflammatory responses. This is supported by intraoperative anterior chamber depth instability and a markedly elevated interleukin-8 (IL-8) concentration (557.3 pg/mL) in the anterior chamber fluid. This case provides *in vivo* visualization of zonular abnormalities in a living Asian PEX patient using endoscopic technology, offering insights that extend beyond the capabilities of traditional imaging. It contributes direct evidence to enhance understanding of the pathogenesis of PEX-related glaucoma and suggests that intraoperative endoscopic assessment may serve as a valuable tool for evaluating zonular integrity and intraocular inflammation in PEX patients. Future integration of artificial intelligence-based image analysis, combined with intraocular fluid inflammatory marker assessment, may enable quantification of deposit parameters and establishment of a zonular lesion grading system, potentially optimizing PEX management.

## Introduction

Pseudoexfoliation syndrome (PEX), a systemic fibroproteinopathy, principally targets the ocular anterior segment. It is marked by the deposition of grayish-white fibrillar substances on the lens capsule, zonules, and other ocular components (1). However, global epidemiological data on PEX remain limited. Current literature findings display pronounced disparities, chiefly attributable to variations in study designs, regional disparities, and sample selection biases. Some prior investigations have spotlighted that females are at a substantially elevated risk of contracting PEX compared to males, and this gender gap becomes even more conspicuous within the elderly female demographic (2). Additionally, another body of research has posited a robust positive association between age and PEX occurrence: concretely, the incidence climbs non-linearly as age advances, peaking at approximately 28% among those aged 75–79 and surging to 30% for individuals 85 years old and older (3). From a geographical perspective, PEX exhibits a latitudinal gradient pattern, with regions at lower latitudes reporting a comparatively lower prevalence, hinting at the involvement of environmental elements in its etiology (4). Because the genuine incidence of PEX within the general population remains elusive and significant discrepancies exist concerning age, gender, and geographical location, case studies can play a pivotal role. By collating the disease courses and ocular pathological manifestations of diverse patients, they can provide direct evidence for unmasking disease heterogeneity, refining early diagnostic benchmarks, and investigating environmental causative mechanisms.

PEX is an age-related disease in which abnormal substances accumulate in the anterior segment of the eyeball. The origin of pseudoexfoliation material is associated with dysregulation during elastin synthesis and the formation of abnormal elastic fiber aggregates, accompanied by a significant reduction in collagen fibers (5). There are numerous theoretical mechanisms regarding the development and progression of this disease. However, direct visualization of such deposits *in vivo* in the ciliary region has been restricted. This is because conventional imaging modalities typically struggle to detect the subtle pathological changes that occur in the deep anterior segment.

In this case report, we present an unusual finding observed during endoscopic cataract surgery in a patient with PEX: extensive gray-white, spider web-like fibrillary deposits covering the zonules and ciliary body surfaces. This observation provides direct *in vivo* evidence of fibrillar material accumulation in the ciliary apparatus, a region critical for maintaining zonular homeostasis and aqueous humor dynamics. The morphology and distribution of these deposits suggest a potential pathogenic role in the initiation or progression of PEX-related structural degeneration.

Although prior histopathological studies have described similar fibrillar accumulations in postmortem eyes (6), our endoscopic documentation underscores the feasibility of visualizing these lesions in living patients. This finding not only expands the spectrum of PEX-associated anterior segment pathology but also highlights the need for further investigations to elucidate the molecular composition and functional implications of these deposits. By linking *in vivo* imaging findings to established pathological mechanisms, this case contributes novel insights into the pathophysiology of PEX and may inform future strategies for early diagnosis and targeted therapies.

## Case report

**Figure 1:**
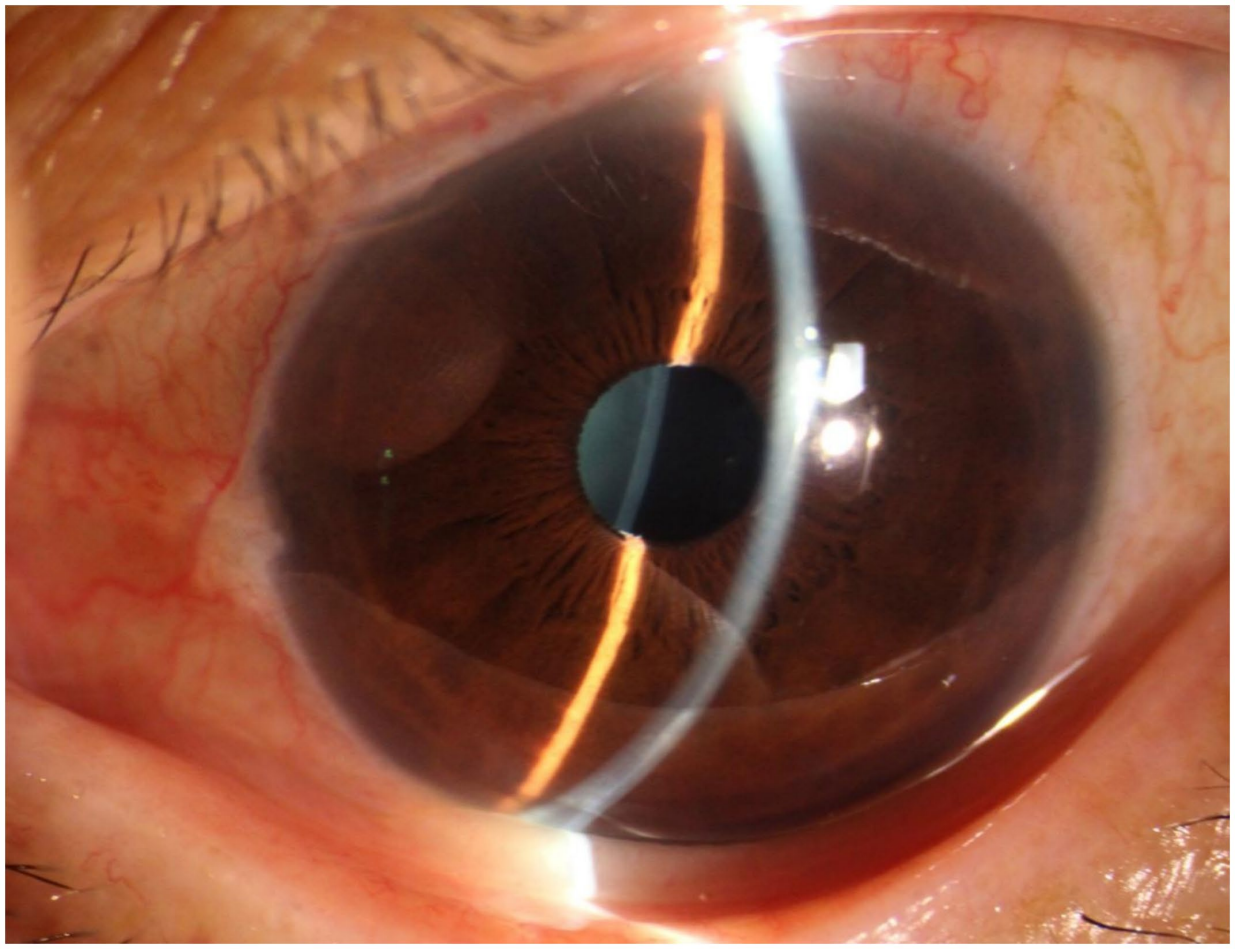
Anterior segment of the patient’s left eye. It can be observed that the anterior chamber depth is not shallow, and a small amount of fine grayish-white debris is visible on the round pupil.

**Figure 2:**
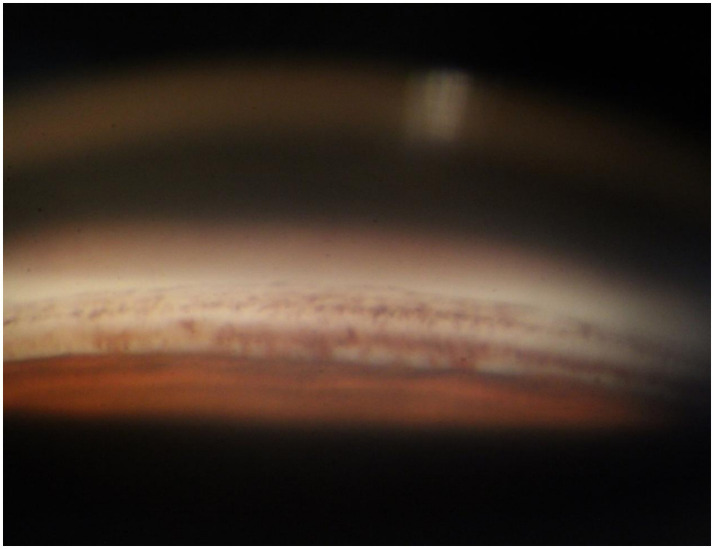
Anterior chamber angle of the patient’s left eye. A large amount of pigment is densely distributed around the trabecular meshwork tissue. These pigments will impair the external filtration function of the trabecular meshwork.

**Figure 3:**
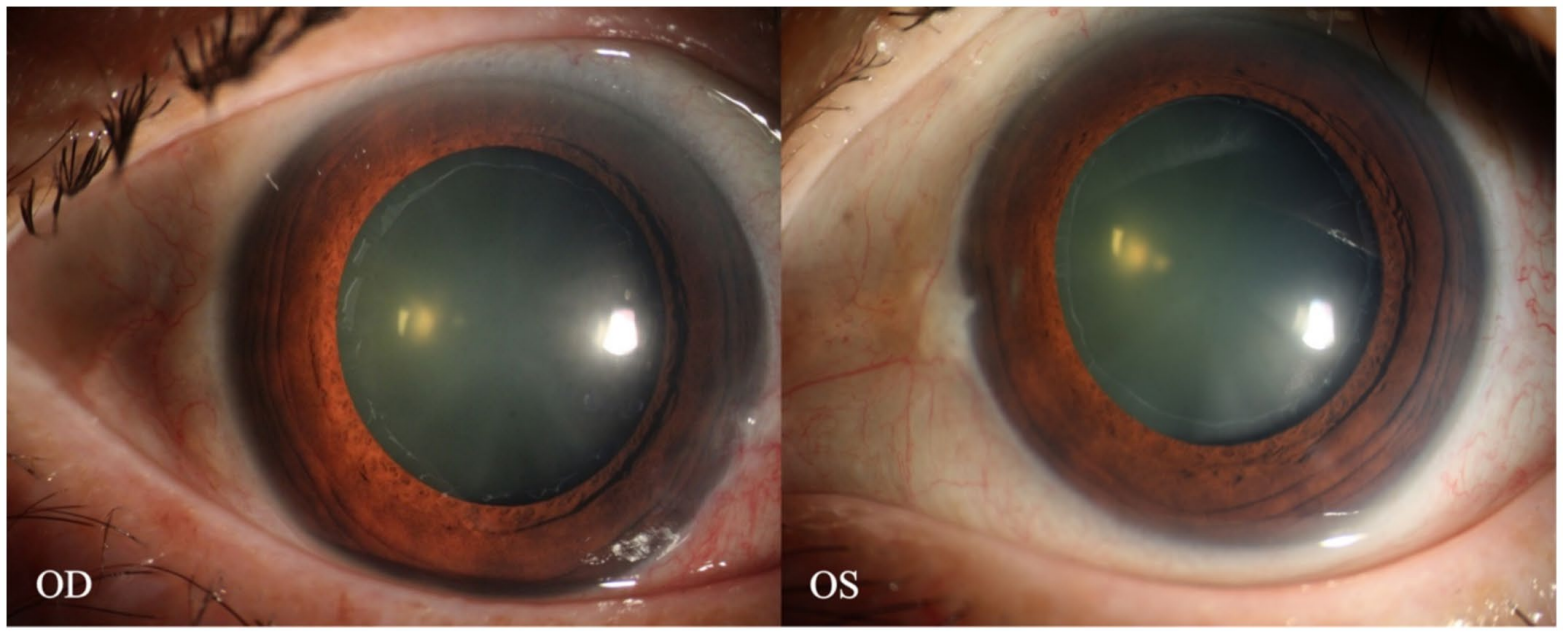
Anterior segment images of the patient’s eyes after pupil dilation. A distinct annular grayish-white opacity zone around the periphery of the anterior lens capsule of both eyes is visible.

A 70-year-old Asian woman presented with left eye soreness and blurred vision for 1 month. When she first visited another hospital during this period, her visual acuity was 0.6 in the right eye and 0.1 in the left eye, with the intraocular pressure (IOP) being 15.2 mmHg in the right eye and 40.1 mmHg in the left eye, and she was diagnosed with left-eye glaucoma. Other ocular conditions at that time were unclear. She was treated with carteolol, brinzolamide, and pilocarpine eye drops for 1 month, but the therapeutic effect was unsatisfactory, so she visited our hospital. When the patient first presented to our hospital, the visual acuity of the right eye was 0.6, with an IOP of 17.1 mmHg, and that of the left eye was 0.6, with an IOP of 26.1 mmHg after using the aforementioned anti-glaucoma medications. The pupils of both eyes exhibited distinct miosis, likely caused by the medications. Considering that the patient was an elderly Asian female with an intumescent cataract, when we first saw her in the outpatient clinic, we initially thought the diagnosis was left-eye angle-closure glaucoma. However, we later discovered that the anterior chamber depths of both eyes were not shallow. It was not until we noticed unequal amounts of grayish-white debris on the pupillary margin of the left eye under the slit lamp (Figure 1). After careful consideration, we performed gonioscopy on the patient under topical anesthesia. We found that the angles of both eyes were open. Although this might have been due to the effect of pilocarpine, we also observed obvious pigment residues in the angles of the eyes (Figure 2). This alerted us to the possibility that the patient might have PEX. Ophthalmic evaluations were performed. Dynamic IOP monitoring revealed fluctuations of 17.1–20.1 mmHg in the right eye and 23.3–28.7 mmHg in the left eye despite treatment with two topical hypotensive agents. Fundus examination showed a red optic disc with a cup-to-disc (C/D) ratio of 0.4 in the right eye, whereas the left eye presented a pale-red optic disc with an increased C/D ratio of 0.7; both eyes had flat retinas with leopard-like fundus changes. Static perimetry (24–2 threshold test) demonstrated a visual field index (VFI) of 96% in the right eye (within normal range) and 66% in the left eye, with a superior arcuate scotoma in the left eye, classic for glaucomatous visual field loss. Optical coherence tomography (OCT) scans further confirmed: (1) in the right eye, no significant thinning of the macular neuroepithelial layer or ganglion cell layer (GCL), with only partial thinning of the retinal nerve fiber layer (RNFL); (2) in the left eye, partial thinning of the macular neuroepithelial layer, marked thinning of the GCL, and significant RNFL thinning—consistent with PEX-related glaucomatous optic neuropathy. Thus, based on the previous examinations, we dilated the pupils of both eyes. The dilation process was markedly difficult, which further strengthened our previous suspicion. Eventually, the anterior segment photographs after pupil dilation of both eyes are shown in Figure 3. We suspected that the development of the events was as follows: The grayish-white flakes on the periphery of the anterior lens capsule act like sandpaper on the iris, facilitating the release of a large amount of pigment from the iris. The released pigment will deposit and clog the trabecular meshwork (5). Upon detailed inquiry, the patient denied any relevant family history.

**Figure 4:**
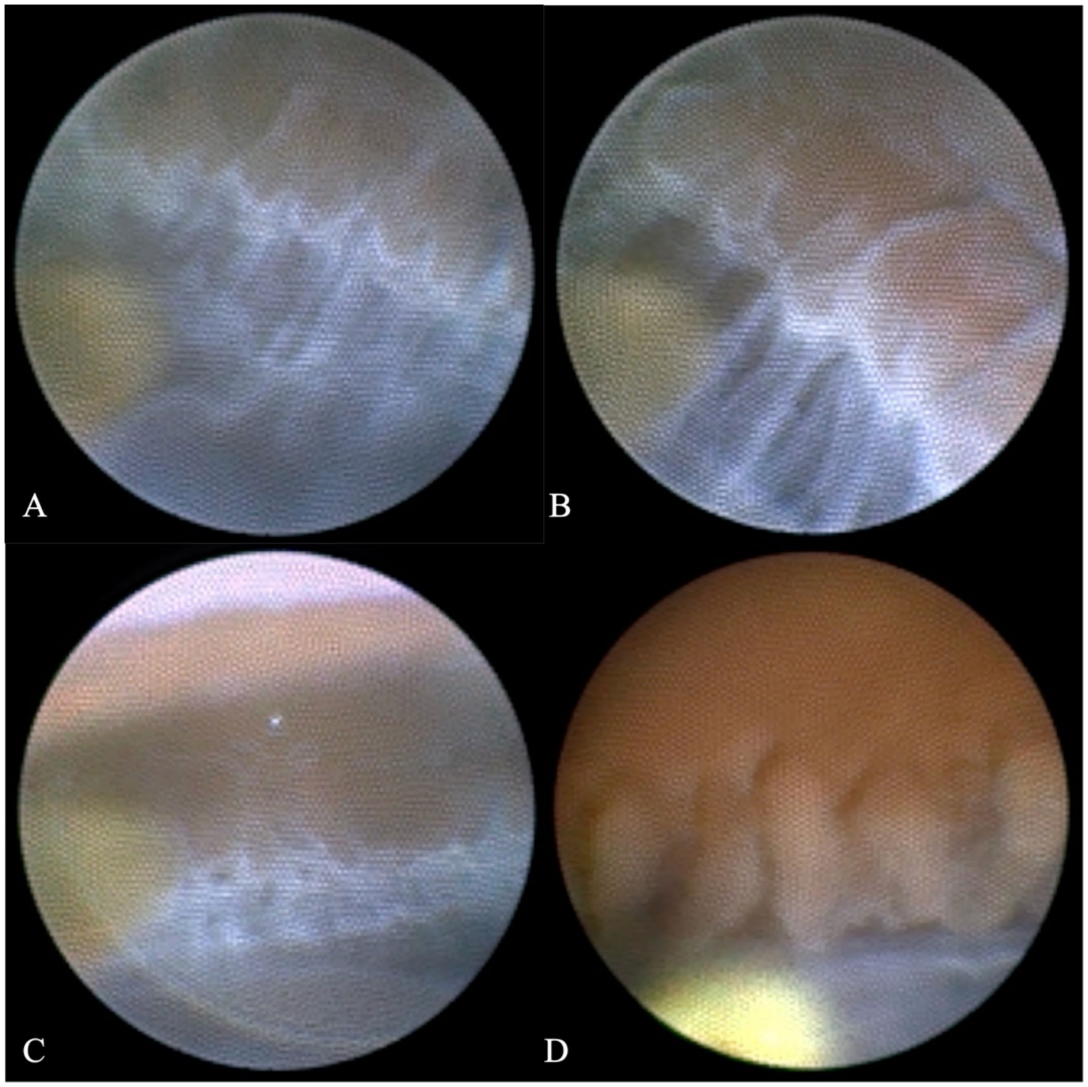
Comparison of the ciliary body and zonules under endoscopy between this patient and other glaucoma patients. Parts **(A)**, **(B)**, and **(C)** of the figure display the ciliary body and zonules from different angles of this patient. The surface of the zonules is covered with a large amount of grayish-white flocculent material, which makes the zonules fully visible. In contrast, part **(D)** of the figure shows that for ordinary glaucoma patients, the ciliary body and part of the capsular bag edge are visible, but the zonules are too transparent to be seen.

Ultimately, we decided to perform cataract extraction surgery first. Given the preoperative evidence of PEX-related glaucomatous changes (e.g., trabecular meshwork pigmentation, left eye RNFL/GCL thinning, and superior arcuate scotoma), the surgical plan was tailored to address both cataract and the underlying PEX pathophysiology. Specifically, by replacing the thick, pseudoexfoliation material-laden natural lens with a thin intraocular lens (IOL), we aimed to: (I) reduce mechanical friction between the iris posterior surface and the lens anterior capsule—an important source of iris pigment shedding in PEX, (II) minimize further pigment detachment and subsequent deposition in the trabecular meshwork, which alleviates aqueous humor outflow resistance, and (III) create a more stable anterior chamber environment to mitigate the risk of intraoperative IOP fluctuations and zonular stress. Additionally, considering the known risk of zonular weakness and dehiscence in PEX patients (7), femtosecond laser-assisted cataract surgery (FLACS) was selected over conventional phacoemulsification to ensure precise capsulotomy and gentle lens fragmentation, thereby reducing intraoperative zonular traction (8, 9). Simultaneously, based on previous studies, we chose to use an endoscope to observe the patient’s zonules during the operation. As shown specifically in Figure 4, we observed that the entire circumference of the zonules was covered with a large amount of grayish-white flocculent material, resembling a spider web. Moreover, during the operation, we distinctly observed unstable changes in the anterior chamber depth, which were consistent with previous research findings. Furthermore, approximately 0.1 mL of anterior chamber fluid was aspirated before the start of the surgery, and quantitative detection of multiple key intraocular cytokines was performed using a flow cytometer to assess the inflammatory profile associated with PEX. Among the detected factors, interleukin-8 (IL-8) was markedly elevated at 557.3 pg/mL—a level consistent with PEX-related chronic inflammatory responses and indicative of neutrophil recruitment, which may contribute to zonular fibrin-like deposits and trabecular meshwork dysfunction. Interleukin-6 (IL-6) showed a slight elevation at 29.8 pg/mL, while all other tested cytokines remained within normal ranges: interleukin-1β: 3.6 pg/mL, interleukin-2: 1.3 pg/mL, interleukin-4: 1.4 pg/mL, interleukin-5: 2.2 pg/mL, interleukin-10: 2.0 pg/mL, interleukin-12p70: 1.6 pg/mL, interleukin-17: 2.3 pg/mL, tumor necrosis factor-α: 1.6 pg/mL, interferon-α: 0.9 pg/mL, interferon-γ: 8.2 pg/mL, and vascular endothelial growth factor: 18.9 pg/mL. This finding confirms that intraocular inflammation in this PEX patient is primarily driven by IL-8 elevation, with minimal involvement of other inflammatory pathways. Accordingly, postoperatively, in addition to the routine use of topical antibiotics, we also administered topical prednisolone eye drops (four times a day) and pranoprofen eye drops (four times a day).

On the first postoperative day, the patient’s visual acuity in the operated eye improved to 1.0. Since anti-glaucoma surgery was not performed, and the patient still had persistent intraocular inflammation in the early postoperative period, leading to unstable IOP at this stage, we continued topical hypotensive therapy with carteolol and brinzolamide, administered twice daily. The IOP was 18.3 mmHg on the first postoperative day and 12.2 mmHg on the second postoperative day, after which the patient was discharged.

At 1-week follow-up, the left eye visual acuity remained 1.0 with an IOP of 12.2 mmHg, so carteolol was discontinued. At 1-month follow-up, the left eye visual acuity was maintained at 1.0, and the IOP measured 14.2 mmHg, prompting the discontinuation of brinzolamide. During the 3-month postoperative review, the visual acuity remained stable at 1.0 with an IOP of 14.6 mmHg without eye drops, and OCT of the left eye showed no further RNFL or GCL thinning. The patient expressed satisfaction, noting that the single surgery addressed both glaucoma and cataract, improved vision and quality of life, and eliminated the need for long-term pressure-lowering eye drops.

## Discussion

It is estimated that up to half of PEX patients will develop glaucoma during their lifetime. Compared with the healthy population, PEX patients have a 6- to 10-fold higher risk of developing glaucoma (5). This implies that PEX patients often require combined glaucoma-cataract surgery. However, in PEX cases, due to the fragility of the zonules and the poor stability of the lens, the risk of intraoperative complications is significantly increased, such as partial zonular rupture, posterior lens capsule rupture, vitreous extension into the anterior segment, and cataract dropping into the vitreous cavity (10, 11).

This study might be the first case to visually demonstrate the abnormal pathological changes in the zonules in a living Asian PEX patient using endoscopic technology. The entire circumference of the zonules was covered with grayish-white flocculent material, and its morphological characteristics were completely consistent with the “fibrin-like deposits” described in autopsy studies (6).

We believe that the exfoliating material scattered on the surfaces of the zonules and ciliary body mainly damages the intraocular environment through the following two major pathways. Firstly, mechanical damage: Fibrin deposition leads to an increase in the surface roughness of the zonules, and the observed fluctuations in the anterior chamber depth during the operation might be related to this. The latest literature confirms that Lysine-like oxidase-1 gene polymorphism is closely associated with the onset of PEX. Single-nucleotide polymorphisms such as rs1048661, rs3825942, and rs11638944 are important risk factors for PEX (12, 13). Their expression can disrupt the stability of collagen cross-linking, making the zonules more prone to rupture. The intraoperative instability of the zonules in this patient was consistent with this mechanism (14).

Secondly, chronic inflammatory response: A large amount of exfoliate material acts as a persistent antigen to stimulate the zonules and ciliary body (15–17). The latest literature reports have found that chemokines such as interleukin-8 and monocyte chemoattractant protein-1 are elevated in the aqueous humor of PEX patients (18), which is consistent with the inflammatory factor detection results of the anterior chamber fluid in this study. Fibrin deposition in PEX is associated with chronic inflammatory and fibrotic processes. IL-6 can participate in this pathological process by activating fibroblasts and promoting extracellular matrix deposition (19). Its slight elevation may reflect a state of chronic, low-grade inflammation rather than acute severe inflammation. Meanwhile, as a pro-inflammatory cytokine, IL-6 often exerts a synergistic effect on other inflammatory factors. Abnormal cytokine expression can recruit neutrophils and activate trabecular meshwork cells. This may lead to an increase in resistance to aqueous humor outflow and subsequently induce an increase in intraocular pressure. Together with the intraoperative pigment dispersion in the angle of the eye and iris friction phenomena in this patient, it suggests the existence of a local inflammatory microenvironment.

Notably, the visualization of zonular lesions in this case provides details that are not easily detectable via traditional imaging techniques such as optical coherence tomography and ultrasonic biomicroscopy, providing direct evidence for understanding the pathogenesis of PEX. This is also an important basis for our inference of the pathogenesis in this study. The literature indicates that zonular abnormalities precede the increase in intraocular pressure (15), suggesting that endoscopic assessment, combined with intraocular fluid testing, when performed intraoperatively during cataract or other anterior segment surgeries in PEX patients, could serve as a valuable tool for evaluating zonular integrity and indirectly reflecting the degree of intraocular inflammation. As an adjunctive measure, leveraging the surgical window rather than an independent invasive examination avoids additional invasiveness while providing direct visualization of deep anterior segment structures that are inaccessible via conventional imaging.

In the future, combined with artificial intelligence-based image analysis, it may become feasible to quantify morphological parameters of the deposits (e.g., coverage rate and thickness) and establish a grading standard for zonular lesions, thereby enhancing the clinical utility of intraoperative endoscopic observations in PEX management.

## Conclusion

This case report contributes to the understanding of PEX by providing *in vivo* endoscopic observations of zonular abnormalities. By leveraging endoscopic technology, researchers could directly observe the abnormal changes in zonular fibers within a living PEX patient, thereby transcending the constraints of traditional imaging modalities. Notably, the fibrillar deposits found on the zonules and ciliary body can potentially disrupt the delicate intraocular environment through both mechanical damage and the induction of chronic inflammation. This finding provides direct evidence that may enhance our understanding of the pathogenesis of PEX-related glaucoma. Additionally, the detection of elevated IL-8 levels in the patient’s anterior chamber fluid further supports the involvement of intraocular inflammatory responses in the pathogenesis of PEX, offering complementary evidence to endoscopic observations. Looking ahead, the integration of future AI-based image analysis holds the promise of quantifying deposit parameters with precision (such as coverage rate and thickness) and establishing a comprehensive zonular lesion grading system. Additionally, combined with the analysis of intraocular fluid inflammatory markers, AI-assisted approaches may further enhance the assessment of disease activity and progression in PEX, thereby providing a more holistic understanding of the condition. In essence, this case report adds to the existing body of knowledge on PEX by providing unique *in vivo* endoscopic observations of zonular abnormalities, and these findings may offer preliminary insights that could inform future research into early diagnostic and therapeutic approaches for PEX management.

## Data Availability

The original contributions presented in the study are included in the article/supplementary material, further inquiries can be directed to the corresponding author.
